# lncRNA *TUSC7* sponges *miR-10a-5p* and inhibits BDNF/ERK pathway to suppress glioma cell proliferation and migration

**DOI:** 10.18632/aging.204655

**Published:** 2023-04-14

**Authors:** Runhui Wang, Jia Wang, Yuanyu Wang, Liang Yang

**Affiliations:** 1 Department of Neurosurgery, Huabei Petroleum Administration Bureau General Hospital, Shijiazhuang, Hebei, China; 2 Department of Neurosurgery, The Second Hospital of Hebei Medical University, Renqiu, Hebei, China

**Keywords:** TUSC7, miR-10a-5p, lncRNA, cerebral gliomas

## Abstract

Objective: Gliomas as primary cerebral malignancies frequently occurring in adults have relatively high morbidity and mortality. The underlying role of long non-coding ribonucleic acids (lncRNAs) in malignancies has attracted much attention, among which tumor suppressor candidate 7 (*TUSC7*) is a novel tumor suppressor gene whose regulatory mechanism in human cerebral gliomas remains inconclusive.

Methods and results: In this study, bioinformatics analysis indicated that *TUSC7* could specifically bind to microRNA (miR)-10a-5p, and according to quantitative polymerase chain reaction (q-PCR), *miR-10a-5p* was up-regulated in human glioma cells and negatively correlated with *TUSC7* expression. Dual-luciferase reporter gene assay showed the ability of *TUSC7* to bind to *miR-10a-5p*, and overexpression of *TUSC7* notably inhibited *miR-10a-5p* expression, restrained human glioma cell proliferation and migration, and regulated cell cycle and cyclin expression via the brain-derived neurotrophic factor/extracellular signal-regulated kinase (BDNF/ERK) pathway. The inhibitory effect of *TUSC7* on *miR-10a-5p* was also verified by designing *miR-10a-5p* overexpression and knockdown panels for wound healing, Transwell and Western blotting assays.

Conclusions: *TUSC7* suppresses human glioma cell proliferation and migration by negatively modulating *miR-10a-5p* and inhibiting the BDNF/ERK pathway, thus acting as a tumor suppressor gene in human gliomas.

## INTRODUCTION

Gliomas are the most commonly identified primary cerebral tumors, of which the incidence rate accounts for 30-40% of central nervous system (CNS) malignancies in adults [1]. They are generally derived from glial cells or precursor cells and then develop into astrocytoma, oligodendroglioma, glioblastoma, etc. [2]. Four grades are summarized in accordance with the degree of malignancy, of which the most aggressive is glioblastoma multiforme (GBM) [3]. The median survival time is short in patients with GBM, only about 15 months, and the 5-year survival rate is only 5% [4]. In spite of comprehensive intervention with surgical resection, radiotherapy and chemotherapy, the prognosis is less satisfactory in nearly all patients, which is attributed to the high malignancy of GBM and the blood-brain barrier. The latter refers to a defensive barrier constituted by vascular endothelial cells, capillaries and basement membrane, which impedes many drugs from entering the brain to exert effects and poses challenges for treating gliomas [5]. Hence, there is a necessity to explore new early diagnostic biomarkers, thus improving the current status of diagnosis and treatment of gliomas.

Driven by surging development and extensive use of ribonucleic acid (RNA) sequencing and bioinformatics techniques, long non-coding RNAs (lncRNAs) have been identified to be involved in numerous biological processes of cancers [6]. Not only participating in tumor occurrence and progression and exerting crucial effects in targeted therapy and prognosis but also acting as tumor promoters or suppressors in various types of cancers, lncRNAs are generally acknowledged as potential biomarkers in cancers [7]. Despite the fact that many lncRNAs (e.g., *TUG1* and *MIR22HG*) have been revealed to be markedly dysregulated in gliomas [8, 9], the functions and mechanisms of lncRNAs in gliomas have not yet been explored thoroughly.

In this study, the expression profiles of lncRNAs were analyzed via bioinformatics approaches, and lncRNA tumor suppressor candidate 7 (*TUSC7*), which was down-regulated in gliomas, was identified from RNA-seq. Low expression of *TUSC7* was associated with poor prognosis in patients with gliomas and could target microRNA (miR)-10a-5p. In addition, the effect of overexpressing or silencing *TUSC7* on glioma cells was revealed by wound healing and Transwell assays, and the potential mechanism by which lncRNA *TUSC7* suppressed glioma progression via sponging *miR-10a-5p* and inhibiting brain-derived neurotrophic factor/extracellular signal-regulated kinase (BDNF/ERK) pathway was investigated, serving as the theoretical foundation and basis for research on gliomas.

## RESULTS

### Screening of DEGs

**Figure 1:**
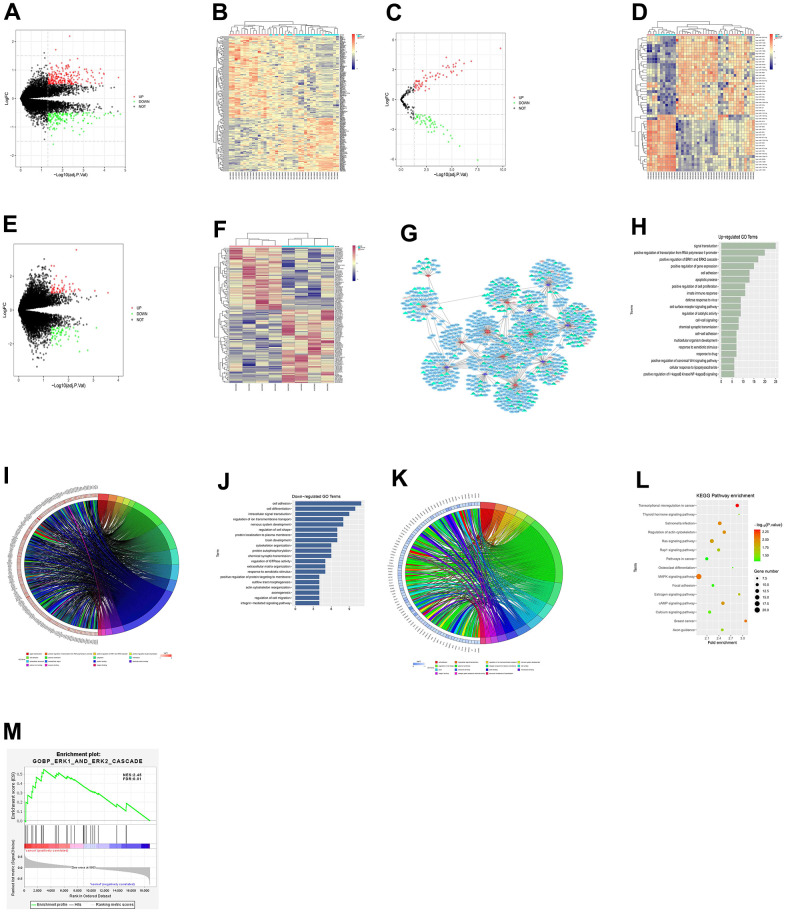
**Results of bioinformatics analysis.** (**A**) Volcano plot of visually grouped DEGs in the dataset GSE57957. (**B**) Cluster heatmap of DEGs. (**C**) Volcano plot of visually grouped DEMs. (**D**) Cluster heatmap of DEMs. (**E**) Volcano plot of visually grouped DELs. (**F**) Cluster heatmap of DELs. (**G**) ceRNA regulatory network map of gliomas. (**H**–**K**) GO pathway diagrams. (**L**) KEGG pathway diagram. (**M**) GSEA for DEGs. ERK1_and_ERK2_cascade and other pathways were identified to be significantly associated enriched pathways.

The glioma-related dataset GSE188256 was downloaded from the GEO database, and the DEGs among samples were analyzed. According to the criteria of *p*-value<0.05 and |logFC|>1, 391 DEGs were found in glioma mRNAs, including 225 up-regulated DEGs and 166 down-regulated DEGs. The volcano plot (Figure 1A) of visually grouped DEGs in the dataset GSE57957 was constructed using the R language ggplot2 software package, and the cluster heatmap (Figure 1B) of DEGs was drawn using the pheatmap software package of R. Using the same method, the volcano plot (Figure 1C) of visually grouped differentially expressed miRNAs (DEMs) and the cluster heatmap (Figure 1D) of DEMs in the dataset GSE158284, as well as the volcano plot (Figure 1E) of visually grouped differentially expressed lncRNAs (DELs) and the cluster heatmap (Figure 1F) of DELs in the dataset GSE83511 were drawn.

### Construction of lncRNA-miRNA-mRNA ceRNA network

To better understand the role of DE-lncRNAs in gliomas, the lncRNA-miRNA-mRNA ceRNA network was constructed. The miRNA-targeted mRNAs were predicted using miRTarBase and TargetScan. Fifteen specific miRNAs associated with 132 interacted mRNAs were found. The targets of some of these genes are cancer-related genes, including BDNF, NTRK2, and MAPK3. By integrating 51 DE-lncRNAs, 12 DE-miRNAs and 132 DE-mRNAs, the ceRNA regulatory network of gliomas was established, and the ceRNA network map (Figure 1G) was drawn using Cytoscape 3.0.

### Bioinformatics analysis

GO and KEGG enrichment analyses were performed on DEGs obtained from the dataset GSE188256. The online database tool DAVID (https://david.ncifcrf.gov) was utilized for DEG analysis from three levels of biological processes, cellular components and molecular functions to integrate GO terms and create a biological process network of DEGs. The GO pathway diagrams (Figure 1H–1K) and the KEGG pathway diagram (Figure 1L) of DEGs were drawn using the R language. As illustrated in GO and KEGG pathway diagrams, both up-regulated pathways (such as positive regulation of ERK1 and ERK2 cascade and positive regulation of cell proliferation) and down-regulated pathways (such as cell adhesion and cell differentiation) were enriched pathways for glioma. Furthermore, through gene set enrichment analysis of GSEA, 109 gene set were selected with criteria FDR <0.05. We also found biological process gene set ERK1_AND_ERK2_CASCADE was significantly associated up-regulated genes (NES=2.45, FDR = 0.01), which was highly consistent with GO enrichment analysis result (Figure 1M).

### *TUSC7* could specifically bind to *miR-10a-5p* in glioma cells

**Figure 2:**
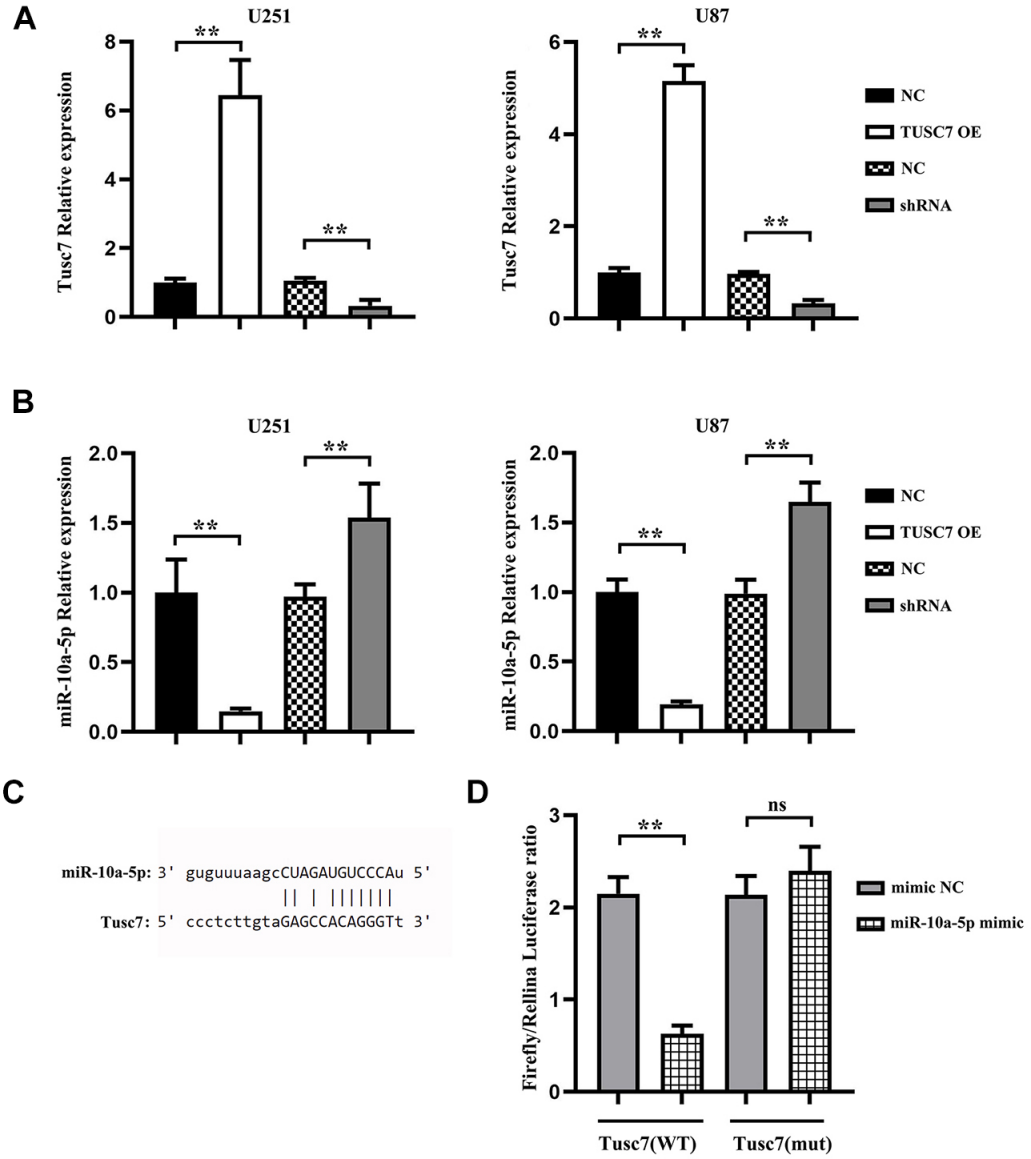
***miR-10a-5p* was the target gene of *TUSC7*.** (**A**) After transfection with *TUSC7* overexpression plasmid and shRNA plasmid, q-PCR was used to measure transfection efficiency. (**B**) Expression of *miR-10a-5p* after overexpression and silencing of *TUSC7* in U251 and U87 cells. (**C**) Binding site of *miR-10a-5p* in the *TUSC7* transcript. (**D**) The results of dual-luciferase reporter gene assay showed that the relative luciferase activity was inhibited after co-transfection of wild-type (WT) plasmids and *miR-10a-5p* mimics, while it was not inhibited after transfection of mutant vector (MUT). ^**^p<0.01; ns: *p*>0.05.

The potential targeting miRNAs of *TUSC7* were predicted using NCBI and jefferson.edu, and the binding site of *miR-10a-5p* in the *TUSC7* transcript was identified. In this study, *TUSC7* overexpression plasmid and shRNA plasmid were transfected into U251 and U87 cells, and the transfection efficiency was measured by q-PCR (Figure 2A). Thereafter, the results of q-PCR manifested that the expression level of *miR-10a-5p* was notably lower in *TUSC7* overexpression group than that in NC group, while it was raised in *TUSC7* silencing group (Figure 2B). Thus, *TUSC7* was hypothesized to repress the proliferation and migration of glioma cells by specifically binding to intracellular *miR-10a-5p*. Furthermore, the luciferase reporter gene assay was used to validate the binding of *TUSC7* to *miR-10a-5p* in glioma cells. In the case that *TUSC7* wild-type and mutant plasmids were co-transfected with *miR-10a-5p* mimic and mimic NC into U251 cells, it was discovered that *TUSC7* wild-type plasmids could specifically bind to *miR-10a-5p* mimic (Figure 2C). These findings suggested that *TUSC7* could target *miR-10a-5p*.

### Overexpression of *TUSC7* suppressed proliferation of glioma cells

**Figure 3:**
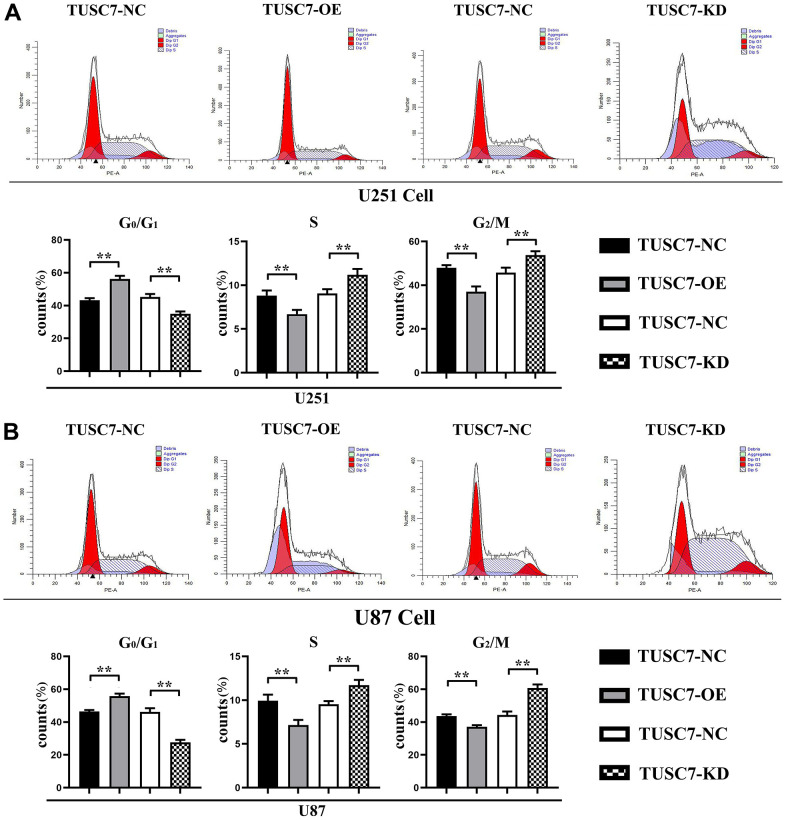
**Overexpression of *TUSC7* suppressed proliferation of glioma cells.** (**A**) Cell cycle assay results of U251 cells. (**B**) Cell cycle assay results of U87 cells. The results of cell cycle assay by flow cytometry showed that compared with NC group, the G1 phase of U251 and U87 cells was significantly increased and the S+G2 phase of cells was significantly decreased when *TUSC7* was overexpressed, and after silencing *TUSC7*, the G1 phase of U251 and U87 cells was significantly decreased, and the S+G2 phase of cells was significantly increased. ^**^p<0.01.

In order to confirm whether malignant biological behaviors such as proliferation of glioma cells were influenced by *TUSC7*, flow cytometry was used to detect the cell cycle. The results showed that compared with NC group, when *TUSC7* was overexpressed, the G1 phase of U251 and U87 cells was significantly increased, and the S+G2 phase of cells was significantly decreased. After silencing *TUSC7*, U251 and U87 cells in the G1 phase were decreased. The results showed that the overexpression of *TUSC7* significantly blocked the cells in the G1 phase, thereby inhibiting the proliferation of U251 and U87 cells (Figure 3).

### Overexpression of *TUSC7* suppressed migration of glioma cells

**Figure 4:**
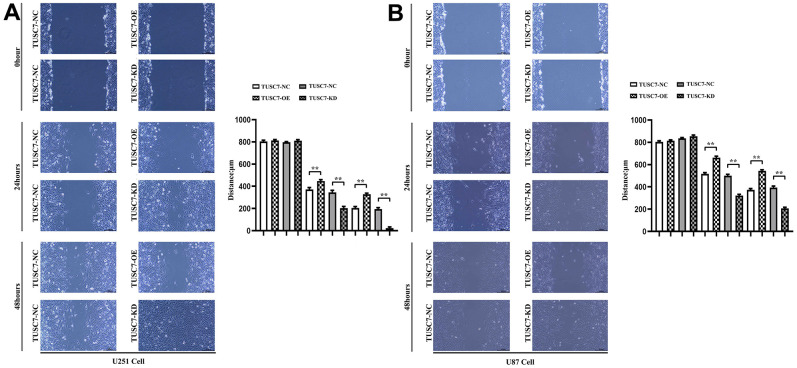
**Overexpression of *TUSC7* suppressed migration of glioma cells.** (**A**) Wound healing assay results of U251 cells. (**B**) Wound healing assay results of U87 cells. The results of wound healing assay displayed that overexpression of *TUSC7* dramatically suppressed the wound healing rate of U251 and U87 cells, whereas it was obviously improved after silencing *TUSC7*. ^**^p<0.01.

**Figure 5:**
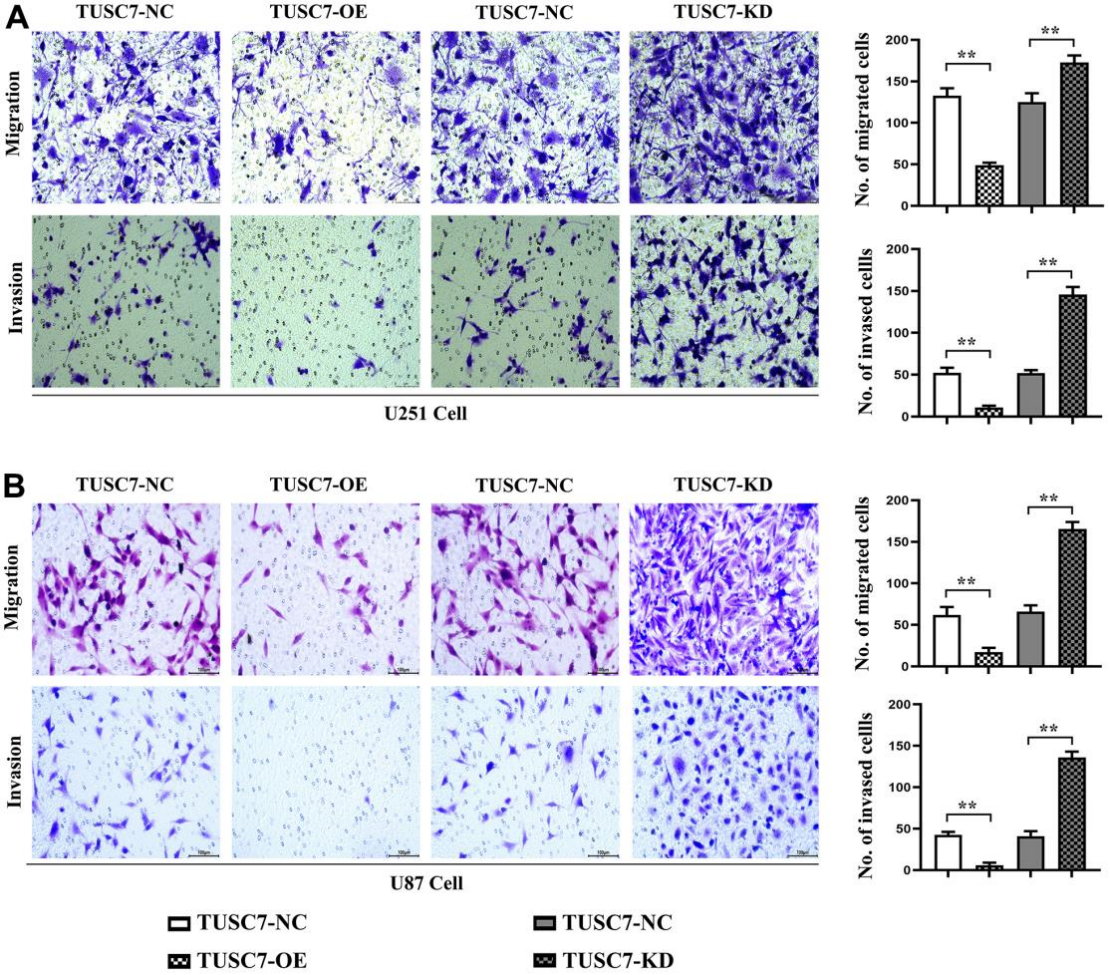
**Overexpression of *TUSC7* suppressed migration and invasion of glioma cells.** (**A**) Transwell assay results of U251 cells. (**B**) Transwell assay results of U87 cells. The results of Transwell assay denoted that overexpression of *TUSC7* distinctly inhibited the migration and invasion abilities of U251 and U87 cells compared with NC group, while they were evidently enhanced after silencing *TUSC7*. ^**^p<0.01.

The results of wound healing assay displayed that overexpression of *TUSC7* dramatically suppressed the wound healing rate of U251 and U87 cells compared with NC group, whereas it was obviously improved after silencing *TUSC7* (Figure 4). The results of Transwell assay denoted that overexpression of *TUSC7* distinctly inhibited the migration and invasion abilities of U251 and U87 cells compared with NC group, while they were evidently enhanced after silencing *TUSC7* (Figure 5). These findings suggested that overexpression of *TUSC7* could significantly suppress the migration ability of glioma cells, whereas the opposite was present after silencing the expression of *TUSC7*.

### Overexpression of *TUSC7* suppressed expressions of cyclin and MMPs in glioma cells

**Figure 6:**
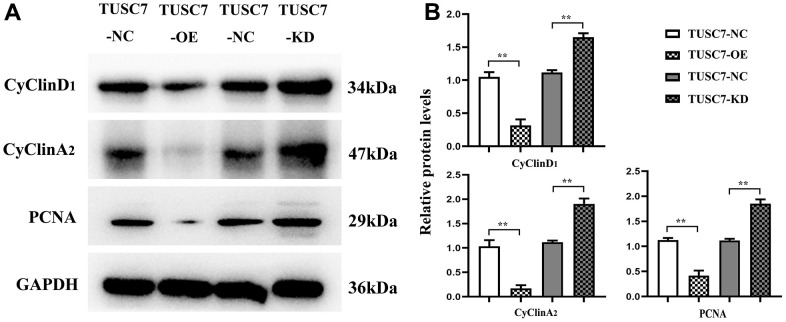
**Overexpression of *TUSC7* suppressed expressions of cyclin in glioma cells.** (**A**) Protein bands of cyclin. (**B**) Relative protein expressions of cyclin. The results of Western blotting revealed that after overexpression of *TUSC7*, the expressions of cyclinD1, cyclinA and PCNA apparently declined compared with those in NC group, whereas they were overtly increased after silencing *TUSC7*. ^**^p<0.01.

CyclinD1 and cyclinA mainly act as promotors for cell proliferation, and PCNA serves as a vital player in the initiation of cell proliferation, which can well reflect cell proliferation status. The results of Western blotting revealed that after overexpression of *TUSC7*, the expressions of cyclinD1, cyclinA and PCNA apparently declined compared with those in NC group, whereas they were overtly increased after silencing *TUSC7* (Figure 6), suggesting that *TUSC7* could suppress the proliferation of glioma cells via cell cycle arrest.

**Figure 7:**
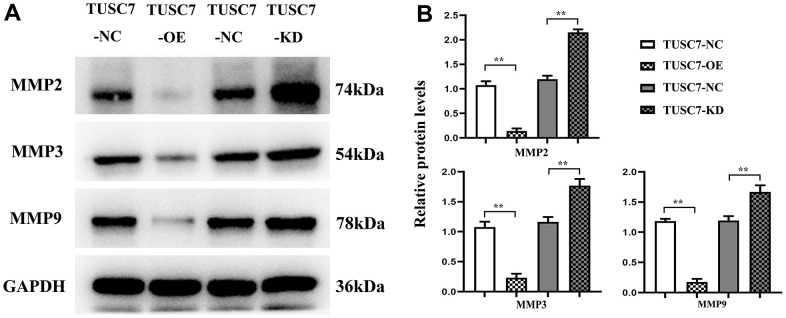
**Overexpression of *TUSC7* suppressed expressions of MMPs in glioma cells.** (**A**) Protein bands of MMPs. (**B**) Relative protein expressions of MMPs. Western blotting results showed that *TUSC7* overexpression group had significantly lower expressions of MMP2, MMP3 and MMP9 than NC group, whereas they were obviously elevated after silencing *TUSC7*. ^**^p<0.01.

MMPs exert important effects in tumor invasion and metastasis, among which MMP2, MMP3 and MMP9 are the main members that play crucial roles in the invasion and metastasis of cancer cells. MMP2 and MMP3 can degrade multiple components of basement membrane and extracellular matrix, and MMP9 is able to induce tumor cells to infiltrate and metastasize into adjacent normal tissues through the injured basement membrane [10]. According to Western blotting results, *TUSC7* overexpression group had significantly lower expressions of MMP2, MMP3 and MMP9 than NC group, whereas they were obviously elevated after silencing *TUSC7*, suggesting that *TUSC7* could inhibit the migration of glioma cells by degrading MMP2, MMP3 and MMP9 (Figure 7).

### Overexpression of *TUSC7* suppressed proliferation and migration of glioma cells by inhibiting the BDNF/TrkB/ERK pathway

**Figure 8:**
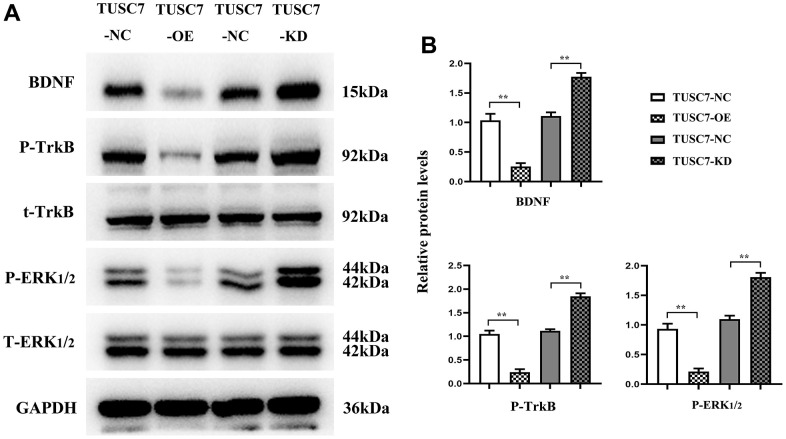
**Overexpression of *TUSC7* could inhibit the expressions of BDNF/TrkB/ERK pathway-related proteins.** (**A**) BDNF/TrkB/ERK pathway-related protein bands. (**B**) Relative expressions of BDNF/TrkB/ERK pathway-related proteins. Western blotting revealed that *TUSC7* overexpression group displayed remarkably lower expression levels of BDNF, p-TrkB and ERK1/2 than NC group, whereas they were transparently raised after silencing *TUSC7*. ^**^p<0.01.

It was recently evidenced that BDNF and its receptor TrkB played significant roles in tumor pathology [11]. Western blotting revealed that *TUSC7* overexpression group displayed remarkably lower expression levels of BDNF, p-TrkB and ERK1/2 than NC group, whereas they were transparently raised after silencing *TUSC7*, suggesting that *TUSC7* could suppress the proliferation and migration of glioma cells through the BDNF/TrkB/ERK pathway (Figure 8).

### Overexpression of *miR-10a-5p* promoted proliferation and migration of glioma cells by activating the BDNF/TrkB/ERK pathway

**Figure 9:**
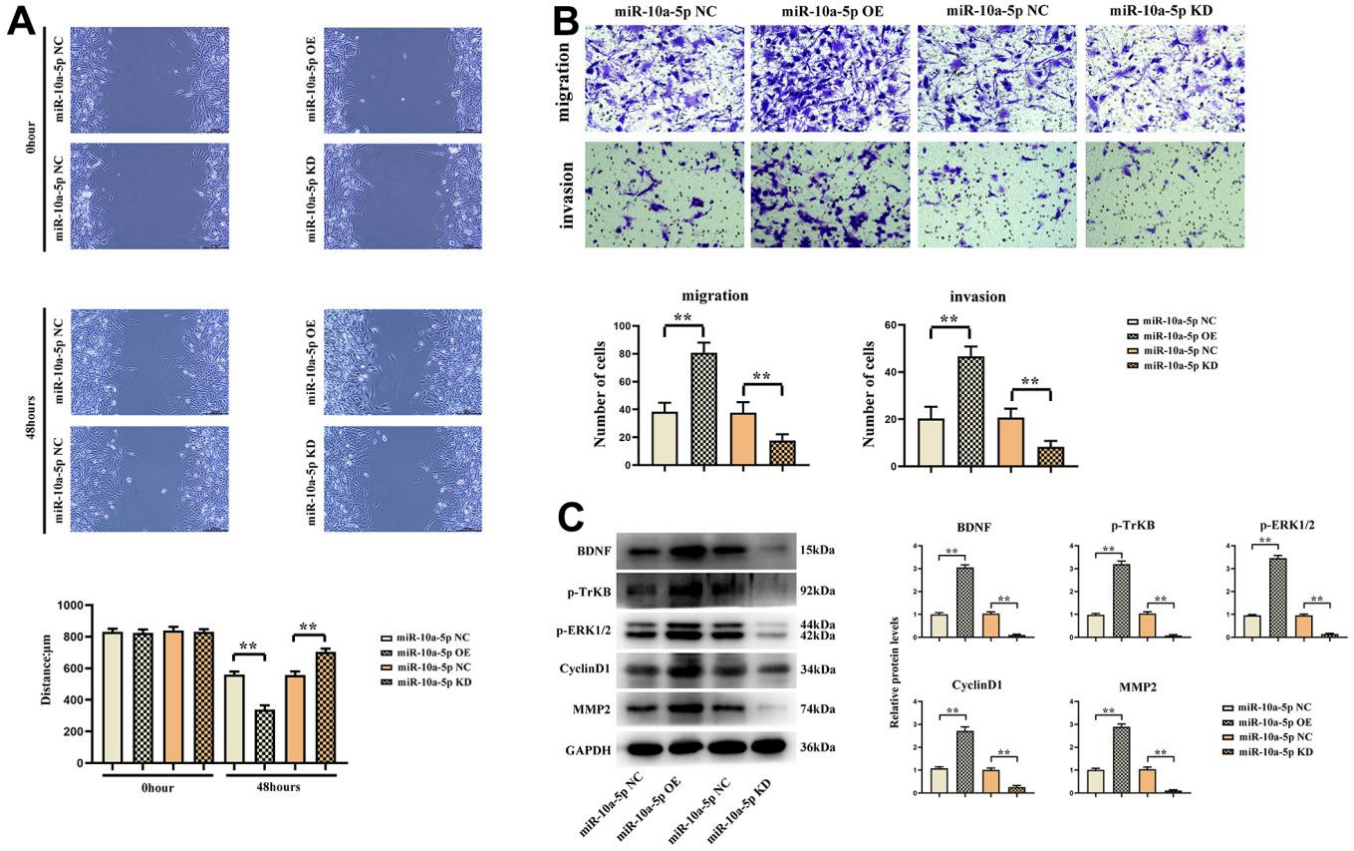
**Overexpression of *miR-10a-5p* could promote the proliferation and migration of glioma cells by activating the BDNF/TrkB/ERK pathway.** (**A**) Wound healing assay results of U251 cells and data statistics at 0 h and 48 h. (**B**) Transwell assay results of U251 cells and data statistics. (**C**) Relative expressions of BDNF/TrkB/ERK pathway-related proteins.

The results of wound healing assay showed that the scratches in *miR-10a-5p*-OE group were significantly smaller than those in NC group at 48 h, and the scratches in *miR-10a-5p*-KD group were significantly larger than those in NC group. Transwell assay results indicated that the number of cells in *miR-10a-5p*-OE group was significantly more than that in NC group, and the number of cells in *miR-10a-5p*-KD group was significantly less than that in NC group. The results of Western blotting revealed that the expression levels of BDNF, p-TrkB and ERK1/2 in *miR-10a-5p* overexpression group were significantly higher than those in NC group, while they were significantly reduced after silencing *miR-10a-5p* (Figure 9).

**Figure 10:**
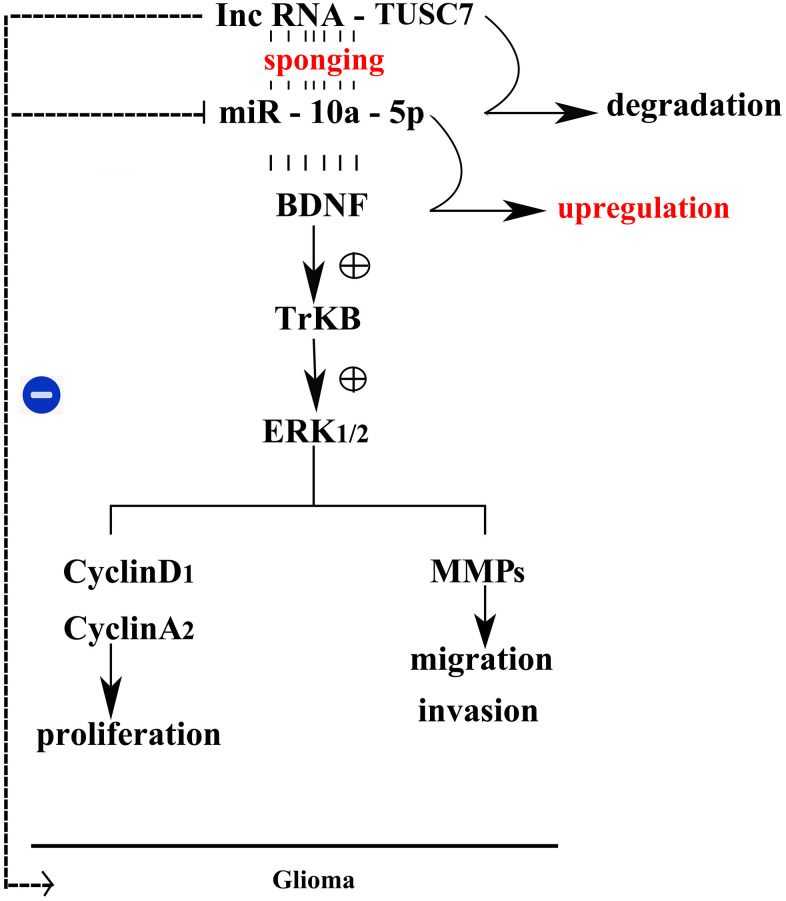
Mechanism schematic diagram of the effects of lncRNA *TUSC7* on gliomas.

The above results showed that *TUSC7* could suppress the proliferation and migration of glioma cells by inhibiting *miR-10a-5p* and mediating the BDNF/TrkB/ERK pathway (Figure 10).

## DISCUSSION

As science and technology develop, great strides have been made in the treatment of cancers. Nonetheless, gliomas, especially GBM, remain one of the most aggressive cancers. Numerous studies on the role of lncRNAs in glioma biology have emerged in recent years, and some lncRNAs may serve as biomarkers for glioma diagnosis, prognosis and drug resistance prediction. However, the correlations of lncRNAs with miRNAs, mRNAs and proteins have not been fully clarified, and the mechanism of their dysregulation in gliomas remains uncertain. *TUSC7* gene, located in chr3q13.31, is 2,105 nt in length and composed of four exons [12]. It was first identified by Pasic et al. from osteosarcoma and has received great attention as a suppressor of human osteosarcoma [13]. *TUSC7* has been proven to be down-regulated in multiple tumors, such as liver cancer [14], colorectal cancer [15], and endometrial cancer [16]. In this study, bioinformatics approaches were first used to screen out DEGs in gliomas, the lncRNA-miRNA-mRNA ceRNA network was constructed, then relevant enriched pathways such as ERK1_and_ERK2_cascade were identified, and *TUSC7* overexpression plasmid and shRNA plasmid were transfected into glioma U251 and U87 cells, followed by investigating the effects of *TUSC7* on malignant biological behaviors of glioma cells. The results suggested that overexpression of *TUSC7* could suppress the proliferation, migration and invasion of U251 and U87 cells. It was speculated that *TUSC7* may exert important effects in the occurrence and progression of gliomas and possibly functions as a tumor suppressor gene.

MiRNAs can modulate gene expressions at the post-transcriptional level, and lncRNAs can act as a “sponge” for miRNAs to reversely regulate gene expressions [17]. Based on biological information mining, the biological effect that *TUSC7* can sponge *miR-10a-5p* has been discovered, in agreement with the reports of Shang et al. [18] that, however, mainly focused on the effect of *TUSC7* on inducing glioma resistance to temozolomide (TMZ) chemotherapy. *MiR-10a-5p* is up-regulated and then promotes cell proliferation, migration and invasion in gliomas [19]. The results of this study revealed that the expression level of *miR-10a-5p* was notably down-regulated after overexpression of *TUSC7* but up-regulated after silencing *TUSC7*, suggesting the inhibitory effect between *TUSC7* and *miR-10a-5p*. In order to elaborate the findings, luciferase reporter gene assay was utilized to validate the specific binding of the sequences between *TUSC7* and *miR-10a-5p*. Therefore, it was speculated that *TUSC7* suppressed the proliferation and migration of glioma cells by, at least partially, inhibiting *miR-10a-5p*. The inhibitory effect of *TUSC7* on *miR-10a-5p* was also verified by designing *miR-10a-5p* overexpression and knockdown panels for wound healing, Transwell and Western blotting assays.

BDNF is considered one of the best-characterized neurotrophic factors in the nervous system, mostly secreted by hippocampal neurons and astrocytes [20]. BDNF can exert its biological function through binding to TrkB. Massive studies have demonstrated that BDNF/TrkB not only can induce nervous system damage via multiple pathophysiological stimuli (such as oxidative stress and inflammation) [21–23], but also plays crucial roles in tumor pathology [11]. It has been uncovered that after cells are stimulated, neurotrophic factors and neurotransmitters can trigger ERK cascades, consequently leading to phosphorylation of cytoplasm and nuclear factors, and the ERK pathway has been identified as an effector molecule of BDNF [24]. The expression levels of BDNF, p-TrkB and ERK1/2 markedly decline after overexpression of *TUSC7*, whereas they are evidently raised after silencing *TUSC7*, suggesting that *TUSC7* can suppress the proliferation and migration of glioma cells through inhibiting the BDNF/TrkB/ERK pathway.

Furthermore, the mechanism of *TUSC7* as a potential tumor suppressor remains elusive. As for gliomas, gene therapy has been rarely studied and its clinical application will face many challenges, including but not limited to the situation: due to the complex network of lncRNAs and their target genomes, whether the therapeutic lncRNAs selected will not affect other non-pathogenic genes and cause long-term side effects cannot be guaranteed. In this study, although it has been proven that *TUSC7* acts as a tumor suppressor in gliomas and has the potential to be a biomarker, *in vivo* and *in vitro* experiments are still needed for further validation, thus providing bases for clinical transformation.

## MATERIALS AND METHODS

### Differential gene screening

Glioma-related mRNA dataset GSE188256(Affymetrix Human Gene Expression Array), miRNA dataset GSE158284(Agilent-021827 Human miRNA Microarray V3) and lncRNA dataset GSE83511(Agilent-045142 Human LncRNA V4) were retrieved and downloaded from the Gene Expression Omnibus (GEO) database (https://www.ncbi.nlm.nih.gov/gds/). Using the R language limma software package, mRNA, miRNA and lncRNA data were subjected to quantile normalization and differentially expressed gene (DEG) (|logFC|>1, *p*-value<0.05) analysis. The volcano plot of visually grouped DEGs in the dataset GSE158284 was constructed using the R language ggplot2 software package, and the cluster heatmap of DEGs was drawn using the R software package pheatmap. In the same way, the volcano plot of visually grouped DEGs in the datasets GSE188256 and GSE83511 and the cluster heatmap of DEGs were drawn.

### Construction of lncRNA-miRNA-mRNA ceRNA network

The ceRNA network was based on the associations among mRNAs, miRNAs and lncRNAs. First, differentially expressed mRNAs, miRNAs and lncRNAs were identified, and then miRcode (http://www.mircode.org) was used to predict lncRNA-miRNA interactions. Interactions between differentially expressed miRNAs and mRNAs were predicted using TargetScan (http://www.targetscan.org/) and miRDB (http://www.mirdb.org/). Cytoscape software (version 3.9.1) was used to construct the ceRNA network.

### Function enrichment analysis of DEGs

Gene ontology (GO) and Kyoto Encyclopedia of Genes and Genomes (KEGG) enrichment analyses were performed on DEGs from the dataset GSE188256. Next, the online database tool DAVID (https://david.ncifcrf.gov) was used for DEG analysis from three levels of biological processes, cellular components and molecular functions to integrate GO terms and create a biological process network of DEGs. Then the GO and KEGG pathway diagrams of DEGs were plotted using GOplot and ggplot2 packages in the R language environment.

### Gene set enrichment analysis (GSEA)

Gene Set Enrichment Analysis (GSEA) performed on the differentially expressed genes. Genes were ranked by fold change by ascending order and the collection of gene set were downloaded from MSigDB (http://www.gsea-msigdb.org/). All 9 categories of gene set collections were used in the enrichment analysis including gene ontology genes set (C5) and pathway gene set pathway(C2). R language was then used to draw the GSEA enrichment analysis pathway map.

### Cell culture and processing

Glioma U251 and U87 cell lines were bought from Wuhan Procell Life Science&Technology Co., Ltd. The cell culture system consisted of MEM medium (Gibco: 42360032) + 10% fetal bovine serum (Gibco: 10099-141) + 1% double antibody (Gibco: 15140-122). Cells were incubated in a constant-temperature and constant-humidity incubator (Thermo Scientific: Form 371) with 5% CO_2_ at 37° C. The cells presented with fibroblast-like morphology under the microscope, and upon reaching >80% confluency, they were digested with 0.25% trypsin digestion solution (Gibco: 25200-056) for 2 min and passaged at a ratio of 1:3.

The *TUSC7* negative control (*TUSC7*-NC) plasmid, *TUSC7* overexpression plasmid (*TUSC7*-OE) and short hairpin (*TUSC7*-KD) plasmid, *miR-10a-5p* negative control (*miR-10a-5p*-NC) plasmid, and *miR-10a-5p* overexpression plasmid (*miR-10a-5p*-OE) and short hairpin (*miR-10a-5p*-KD) plasmid were synthesized by Sangon Biotech. Cells were inoculated into a 6-well plate, and when they reached 50-60% confluency, the plasmids were introduced into the cells using Lipofectamine 3000 (Invitrogen, L3000001). Transfection efficiency was measured by quantitative polymerase chain reaction (q-PCR).

### Q-PCR

Primers used in the experiment were synthesized by Sangon Biotech and sequenced as follows: *TUSC7*: forward primer: 5’-GAACCGTGAGCGCATTTCTC-3’, reverse primer: 5’-CAGCCAGATGGGATGGTTGT-3’; *miR-10a-5p*: forward primer: 5’-ACCCTGTAGATCCGAATTTGTGTAA-3’, reverse primer: 5’-AGAGCGGAGTGTTTATGTCAACT-3’. After the trypsin-digested cells were harvested, the total RNA was extracted from samples using TRIzol reagent (Invitrogen: 15596026) and reversely transcribed into cDNA using PrimeScrip RT Master Mix (TaKaRa: RR036A). The cDNA concentration was detected by microspectrophotometer (Thermo Scientific: A51119500C), followed by determination of *TUSC7* and *miR-10a-5p* expression levels via real-time fluorescent q-PCR with TB Green Premix Ex Taq (TaKaRa: RR420A). Internal references were normalized using glyceraldehyde-3-phosphate dehydrogenase (GAPDH), and the relative expression levels of indicators were calculated by 2^-ΔΔCT^ method.

### Dual-luciferase reporter gene assay

A putative *miR-10a-5p* binding site (UGUCCCA) was identified in the 3’-untranslated region (3’-UTR) of *TUSC7* gene using NCBI and jefferson.edu. Wild-type and mutant *TUSC7* plasmids pmirGLO-*TUSC7*-3’-UTR-WT (WT) and pmirGLO-*TUSC7*-3’-UTR-MUT (MUT) were co-transfected with *miR-10a-5p* (NC/mimic) into U251 cells, followed by incubation for 48 h. After cells in each group were harvested and lysed, the fluorescence values of firefly luciferase and renilla luciferase were determined, and the ratio of the two was calculated. In each group, experiments were repeated in three wells.

### Wound healing assay

The transverse lines were drawn on the back of the 6-well plate (at least 5 lines/well) using a ruler, and the transfected cells were digested with trypsin and spread (5×10^5^ cells/well) on the plate. The next day, they were scratched perpendicular to the transverse line on the back using a 1-mL pipette tip, washed with PBS, and added with serum-free medium, followed by sampling at 0, 24 and 48 h and photographing.

### Transwell assay

In the migration assay, the transfected cells were digested with trypsin, whose density was then adjusted to 1×10^6^/mL using serum-free medium. Next, 100 μL of cell suspension was added to the Transwell chamber with polycarbonate membrane (well diameter: 8 μm; Corning: 3422), and 700 μL of complete medium was added to the lower chamber. After 24 h of incubation, the chamber was fixed with paraformaldehyde and stained with 0.1% crystal violet for 10 min. Then the cells on the medial side of the chamber were gently wiped off with cotton swabs, and only the cells on the lateral side of the chamber were retained, followed by photographing under an inverted bright-field microscope. Differing from the procedures in the migration assay, it was required in the invasion assay that a layer of matrigel matrix (Ceturegel™ Matrigel Matrix LDEV-Free: 40183ES08) should be coated into the Transwell chamber prior to cell placement.

### Western blotting assay

After the total proteins were extracted from cells in each group, the protein concentration was detected using the BCA Protein Assay Kit (Beyotime: P0012), and the samples were added with 5× electrophoresis buffer according to the proportion and placed in a metal bath (100° C) for 10 min. Next, the protein samples were loaded (20 μg/well), separated by SDS-PAGE, and transferred onto a PVDF membrane. After being blocked with 5% skim milk powder (OXOID: LP0031B) or 5% Fatty Acid Free BSA (WarBio: M9203), the membrane was incubated with primary antibodies against cyclinD1 (Abcam: ab16663, 1:200), cyclinA2 (Abcam: ab181591, 1:2,000), proliferating cell nuclear antigen (PCNA) (Abcam: ab29, 1:1,000), matrix metalloproteinase 2 (MMP2) (Abcam: ab92536, 1:1,000), MMP3 (Abcam: ab52915, 1:1,000), MMP9 (Abcam: ab76003, 1:1,000), BDNF (Abcam: ab108319, 1:1,000), phosphorylated tropomyosin kinase receptor B (p-TrkB) (Abcam: ab229908, 1:1,000), total TrkB (t-TrkB) (Abcam: ab134155, 1:1,000), p-ERK1/2 (Abcam: ab184669, 1:10,000), t-ERK1/2 (Abcam: ab17942, 1:1,000) and GAPDH (Abcam: ab8245, 1:5,000) at 4° C overnight. The next day, the membrane was rinsed and incubated with the secondary antibody (Abcam: ab97080, 1:5,000) at room temperature for 1 h. Afterwards, the membrane was rinsed and the proteins were visualized using ECL reagent, followed by statistical analysis.

### Data analysis

SPSS 22.0 software was used for data analysis, and quantitative data were described by mean ± standard deviation. Data conforming to normal distribution and homogeneity of variance were compared by paired *t*-test within the group, and by non-paired *t*-test between two groups. One-way analysis of variance followed by Tukey post-hoc test was used for comparison among groups. *p*<0.05 was considered statistically significant.
